# Computational analysis of calcium signaling and membrane electrophysiology in cerebellar Purkinje neurons associated with *ataxia*

**DOI:** 10.1186/1752-0509-6-70

**Published:** 2012-06-15

**Authors:** Sherry-Ann Brown, Leslie M Loew

**Affiliations:** 1 Richard D. Berlin Center for Cell Analysis & Modeling, University of Connecticut Health Center, 400 Farmington Ave, Farmington, CT 06030, USA

**Keywords:** Model, IP3R, Homer, CAG repeat instability, K_Ca_ channels, *Ataxia*, Computational, BK channel, Spinocerebellar, Virtual cell

## Abstract

**Background:**

Mutations in the smooth endoplasmic reticulum (sER) calcium channel Inositol Trisphosphate Receptor type 1 (IP3R1) in humans with the motor function coordination disorders Spinocerebellar *Ataxia* Types 15 and 16 (SCA15/16) and in a corresponding mouse model, the IP3R1^delta18/delta18^ mice, lead to reduced IP3R1 levels. We posit that increasing IP3R1 sensitivity to IP3 in *ataxias* with reduced IP3R1 could restore normal calcium response. On the other hand, in mouse models of the human polyglutamine (polyQ) *ataxias,* SCA2, and SCA3, the primary finding appears to be hyperactive IP3R1-mediated calcium release. It has been suggested that the polyQ SCA1 mice may also show hyperactive IP3R1. Yet, SCA1 mice show downregulated gene expression of IP3R1, Homer, metabotropic glutamate receptor (mGluR), smooth endoplasmic reticulum Ca-ATP-ase (SERCA), calbindin, parvalbumin, and other calcium signaling proteins.

**Results:**

We create a computational model of pathological alterations in calcium signaling in cerebellar Purkinje neurons to investigate several forms of spinocerebellar *ataxia* associated with changes in the abundance, sensitivity, or activity of the calcium channel IP3R1. We find that increasing IP3R1 sensitivity to IP3 in computational models of SCA15/16 can restore normal calcium response if IP3R1 abundance is not too low. The studied range in IP3R1 levels reflects variability found in human and mouse ataxic models. Further, the required fold increases in sensitivity are within experimental ranges from experiments that use IP3R1 phosphorylation status to adjust its sensitivity to IP3. Results from our simulations of polyglutamine SCAs suggest that downregulation of some calcium signaling proteins may be partially compensatory. However, the downregulation of calcium buffer proteins observed in the SCA1 mice may contribute to pathology. Finally, our model suggests that the calcium-activated voltage-gated potassium channels may provide an important link between calcium metabolism and membrane potential in Purkinje cell function.

**Conclusion:**

Thus, we have established an initial platform for computational evaluation and prediction of *ataxia* pathophysiology. Specifically, the model has been used to investigate SCA15/16, SCA1, SCA2, and SCA3. Results suggest that experimental studies treating mouse models of any of these *ataxias* with appropriately chosen peptides resembling the C-terminal of IP3R1 could adjust receptor sensitivity, and thereby modulate calcium release and normalize IP3 response. In addition, the model supports the hypothesis of IP3R1 supersensitivity in SCA1.

## Background

**Figure 1:**
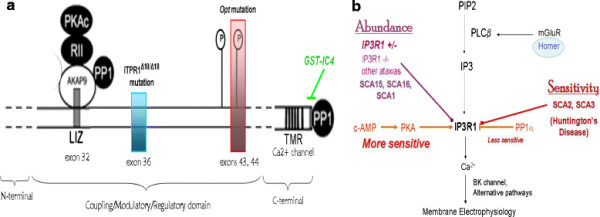
**Schematics relevant to this study.a**, A portion of the regulatory domain of IP3R1 and the C-terminal, adapted from [21,22], with the PP1 binding motif and mutations for ITPR118/18 and ITPR1op1/opt mice. **b**, Summary of various aspects of this study. IP3R1 is the center of this study, involved in phosphoinositol signaling, calcium release, membrane potential modulation; the dysregulation of any of these functions may lead to *ataxia.*

Degeneration of the cerebellum or dysfunction of the Purkinje neurons leads to lack of motor coordination, or *ataxia*, and impaired motor learning [1]. There are several forms of hereditary *ataxia,* but the genetic basis of many *ataxias* is unknown. The IP3R1 calcium channel has been implicated in ataxic mice [2-11] and very recently in humans with *ataxia*[3,9,12-17] (see reviews [18,19]). A portion of IP3R1 is represented in Figure 1a and, along with Table 1, will be referenced in various sections of this computational study, which focuses on a handful of *ataxias* involving this key calcium handling protein. Spinocerebellar ataxia 15 (SCA15) and Spinocerebellar *ataxia* 16 (SCA16) are two forms of autosomal dominant pure cerebellar *ataxia* that involve enormous heterozygous deletions or missense mutations in IP3R1 [15-17] (see Figure 1b and Table 1). However, pathophysiology of SCA15/16 has not been studied extensively. The ITPR1^Δ18/wt^ and ITPR1^Δ18/Δ18^ mice (mouse models for SCA15/16 [3]) possess heterozygous and homozygous, respectively, in-frame 18 bp deletions in exon 36 of the gene that encodes IP3R1. These mice showed reduced levels of cerebellar IP3R1 on Western blot and with immunofluorescence [3] (Figure 1b). IP3R1^+/−^ mice also exhibit motor discoordination on the rotor rod test, and IP3R1^−/−^ mice show lack of balance when upright [2,5]. Various other forms of *ataxia* in mice and humans with causal mutations in genes other than IP3R1 also show reduced IP3R1 protein levels [11,14,20]. Taken together, these findings suggest that haploinsufficiency of IP3R1 may contribute to cerebellar *ataxia*[12].

**Table 1:** Ataxic mouse models relevant to this study

***Ataxia* model**	**Mutated gene product**	**Protein name**	**Pathology**	Compensation *Observed/**Suggested Therapeutic
BK -/-	BK	Large conductance calcium-activated voltage-gated potassium channel	Altered Precision Timing	---
IP3R1 -/-	IP3R1	Inositol triphosphate receptor 1	No IP3R1	--
IP3R1 +/-	IP3R1	Inositol triphosphate receptor 1	R	S^**^
IP3R1 delta18/delta18	IP3R1	Inositol triphosphate receptor 1	R	S^**^
IP3R1 opt/opt	IP3R1	Inositol triphosphate receptor 1	R	Equivocal
SCA15	IP3R1	Inositol triphosphate receptor 1	R	S^**^
SCA16	IP3R1	Inositol triphosphate receptor 1	R	S^**^
PMCA -/-	PMCA	Plasma Membrane Calcium ATPase	No PCMA	R^*^
SCA1	Ataxin1	Ataxin-1	P,A,I	R^*^
SCA2	Ataxin2	Ataxin-2	P,A,S,I	R^**^
SCA3	Ataxin3	Ataxin-3	P,A,S,I	R^*^

P = Polyglutamine expansion; S = Supersensitive IP3R1; A = Association of mutant Ataxin with IP3R1 C-terminal; R = Reduced IP3R1 abundance; I = Increased calcium release.

The mechanism of pathology is different in polyglutamine *ataxias*. Humans with spinocerebellar *Ataxia* 1 (SCA1), spinocerebellar *ataxia* 2 (SCA2), and spinocerebellar ataxia 3 (SCA3), and their corresponding mouse models, have large numbers of unstable polyglutamine (polyQ, CAG) repeats in the Ataxin-1 [23,24], Ataxin-2 [25], and Ataxin-3 [26-28] proteins, respectively. All three mouse models show increased calcium release in response to IP3, relative to wild type mice [6,8,10], with results from SCA2 and SCA3 suggesting a mechanism involving supersensitive IP3R1 [6,10,18]. SCA1 and SCA3 mice have reduced expression of IP3R1 and other glutamatergic signaling proteins [29], in addition to increased IP3R1-mediated calcium release, relative to wild type mice [8]. This paradox is yet to be explained [30]. Gardner suggests that SCA15/16 in humans found to have actual mutations in *ITPR1*, the gene that encodes the IP3R1 protein, be termed *ITPR1-associated ataxia*[31]. However, we have termed a subset of *ataxias,* not just with reduced IP3R1 but also with supersensitive IP3R1, and regardless of the mutated gene (e.g., *ITPR1, Ataxin-1, Ataxin-2, Ataxin-3*) as ‘*IP3R1-associated ataxias*’ [32].

A prior modeling study by Hernjak et al. explores the significance of experimental observations in normal cerebellar Purkinje neurons, which show markedly lower sensitivity and higher abundance of IP3R1 relative to other cell types [33-35]. This is the opposite of what is seen in SCA3 and suggested in SCA1. Hernjak et al. demonstrated that the normally high levels of IP3R1 ensure robust calcium signals in an individual spine, and that the normally low sensitivity of IP3R1 to IP3 confines the calcium transients to the stimulated spine, without spillover to adjacent spines [35]. Thus, they argued that IP3R1 abundance and sensitivity have independent effects on calcium signals and that changes in one do not appear to appropriately compensate for abnormalities in the other. In this work, however, we explore this argument by computationally adjusting IP3R1 sensitivity to IP3 in SCA15/16 (in the context of reduced IP3R1 levels) to see if interventions of this type can actually restore normal calcium response indistinguishable from wild type. We also suggest that, in polyglutamine SCAs with hyperactive IP3R1, experimentally observed downregulation of IP3R1 and other calcium signaling proteins (but not calcium buffers) could serve to partially restore normal calcium transients in *ataxias*.

We test our hypotheses in a novel version of a recently developed computational model that links biochemistry with electrophysiology [36]. We simulate the application of different experimentally available peptides that resemble various portions of the C-terminal of IP3R1, in order to model competitive inhibition of the binding of mutant ataxin proteins. Improvements are made to the model, resulting in an ability to look at changes in membrane potential in response to IP3R1-mediated calcium release or supralinear calcium transients. We show that adjusting IP3R1 sensitivity to IP3, using peptides resembling the C-terminal of IP3R1, could potentially stabilize calcium, without altering fine-tuning of supralinear calcium release required for normal Purkinje neuron function and long term-depression. The model also demonstrates that decreased levels of IP3R1 and various other calcium signaling proteins (except for calcium buffers) experimentally observed in SCA1 mice may partially compensate for the IP3R1 supersensitivity suggested by Liu et al. [10]. Finally, we link IP3R1 activity with the influence of calcium on membrane electrophysiology. Results from our study may provide further insight into the roles that *abundance* and *sensitivity* of the calcium channel IP3R1 play in cerebellar function and coincident detection at IP3R1. Results may also provide a foundation for future studies on SCA1, SCA2, SCA3, SCA15, SCA16, and other IP3R1-associated *ataxias,* which all involve aberrant calcium signaling.

## Methods

### Merged biochemical and electrophysiological modeling

Our analysis of IP3 and calcium dynamics in SCA Purkinje neurons is based on several computational studies of normal physiology from our lab [32,35,36]; these will be briefly described.

The cerebellar Purkinje neuron geometry is complex. In order to combine detailed biochemistry with electrophysiology in tractable Virtual Cell models (VCell; http://www.vcell.org)[37-41], we developed a geometry reduction algorithm *preserved path reduction* or *PPR*[36]. This algorithm simplifies the Purkinje neuron geometry to just the essential dendritic segments and branches in a path between a prototypical dendritic spine and the soma, while maintaining the dynamic electrical features of the full geometry in these respective compartments. The resulting model has only 17 compartments and allows us to readily couple the biochemistry in the spine compartment with the electrophysiology of the entire system. Reference [36] provides full details of the algorithm and the resultant geometry.

The baseline VCell model for normal physiology of the system was also previously published by our laboratory [35]. This compartmental model focused on IP3R1-mediated calcium signaling in the Purkinje cell, taking into account the increased abundance and decreased sensitivity of the Purkinje cell IP3R1 relative to other cell types. The input to the system is provided as a train of IP3 pulses (representing IP3 production from multiple parallel fiber stimuli) with variable parameters that control the number, amplitude and duration of the IP3 pulses. In addition, the model included influx of calcium representing the opening of voltage-gated calcium channels when the Purkinje neuron is stimulated by a climbing fiber. It therefore allowed the study of coincident activation of the Purkinje spine by multiple parallel fibers and a single climbing fiber. The model also included the downstream effects leading to IP3R1-mediated calcium release, as well as calcium buffering and SERCA, which resides on the sER. All of the spine cytosol species are free to diffuse through the spine neck into the adjacent dendrite. IP3 also diffuses in the cytosol to bind its calcium channel-coupled receptor on the sER membrane. Calcium is released from sER stores into the cytoplasm and also co-activates IP3R1, as well as binds to various buffers. Some of this calcium is also pumped back into the sER by SERCA. The model definition in VCell includes specifying reactions, membrane transport mechanisms, stoichiometry, and kinetic rate expressions that correspond with these processes.

We subsequently created a 3D spatial model that explored [42] the membrane signaling mechanisms upstream of IP3R. We distinguished between candidate sources of sufficient phosphatidylinositol-4,5-bisphosphate (PIP2) in the spine for the production of requisite IP3. Local PIP2 sequestration provided an efficient means of fine-tuning coincident activation of parallel fiber and climbing fiber stimuli in cerebellar Purkinje neuron spines. The results reported in this study concluded that local high PIP2 achieved through a sequestration mechanism could provide sufficient IP3 for supralinear calcium release.

We used optimized parameters [42] to study physiological effects of varying IP3R1 sensitivity and abundance with a physiologically appropriate IP3 signal amplitude and duration. To manipulate IP3R1 *abundance* and *sensitivity*, we adjusted the parameters *Jmax* and *d*_IP3_, respectively, in our comprehensive model (see a table of parameters in [42]). Small *Jmax* values represent reduced IP3R1 abundance. The default value is from Hernjak et al. [35], and is 10x larger than the IP3R1 determined for neuroblastoma cells, to account for the increased levels of IP3R1 in Purkinje neurons. The *d*_IP3_ parameter represents the dissociation constant of IP3 from IP3R1, so that high *d*_IP3_ values in our model represent low affinity of IP3R for IP3. We use the apparent affinity of IP3R1 for IP3 as a measure of sensitivity, as in the experimental study by Tang et al. [43]. Manipulation of these IP3R1parameters was done in the context of a train of 4 parallel fiber (PF) stimuli followed by a single climbing fiber (CF) stimulus, following the optimal protocol designed by Wang et al. [44]. This allowed us to examine the effects of IP3R1 insufficiency and supersensitivity in the context of Purkinje spine stimulation patterns similar to those in published experimental studies [35,42,44-46].

We investigate the pathophysiology of various SCAs, as well as the influence of IP3R1-mediated calcium release on the activity of voltage-gated K_Ca_ channels, and thus on membrane potential. We also combine essential features of the spatial and compartmental models to create a 3D model of the spine. We use this model to probe the significance of Homer (associated with the postsynaptic density) physically linking mGluR to IP3R1. The resulting multicompartmental and 3D spatial Virtual Cell models can be accessed in the public domain at http://www.vcell.org under the shared username Brown. The models are:

1. “Brown et al. 2011 Analysis of SCA15-SCA16 with IC4 Peptide Application”,

2. “Brown et al. 2011 SCA1 Compensation Analysis”,

3. “Brown et al. 2011 Analysis of SCA1-SCA2-SCA3 with IC-G2736X Peptide”,

4. “Brown et al. 2011 Purkinje Biochem-Electrophysiol SCA”,

5. “Brown et al. 2011 Purkinje 3D Spine – Spinocerebellar *Ataxia* study”, and

6. “Brown et al. 2011 Combined Purkinje - Several Spines - Current Injection”.

### Adapting the PPR model to the SCA model

In order to investigate how biochemical signaling at the spine could affect electrophysiology, we needed to allow calcium release to influence the voltage-gated K_Ca_ channels. In the PPR model, K_Ca_ channels were only influenced by calcium influx into a phenomenological submembrane shell. This allowed for very high transient concentrations of calcium (500 μM), as well as a high concentration of resting calcium (~ 4 μM) near the inner surface of the plasma membrane. Since such a high amount of basal calcium is not physiologically observed or observable (in the absence of Purkinje neuron stimulation by nerve endings from regulatory cells) and since calcium concentration beyond the submembrane shell was not explicitly considered in the PPR model, we felt it was important to model calcium less phenomenologically. Therefore, in this work, we removed submembrane shell calcium from the model only in the spine and the adjacent dendrite, where calcium signaling biochemistry is modeled explicitly. The adjacent dendrite was adjusted in tandem with the spine, because calcium and other molecules involved in biochemical processes are allowed to diffuse freely between the spine and the adjacent dendrite, restricted only by the geometry of the spine neck. In a previous study, Hernjak et al. [35] used a one-dimensional model to determine a dendritic distance, 28 μm from a stimulated spine, at which biochemical concentrations remained at steady state during synaptic activity. The model therefore assumes that there is no net molecular flow between compartments, except for the adjacent dendrite (given a length of 28 μm) and the spine.

In other compartments, calcium influx feeds only into submembrane shell calcium. In the spine and adjacent dendrite, calcium influx is allowed to directly alter concentration of calcium involved in biochemical signaling; we term this calcium ‘global calcium’. The concept of the submembrane shell in electrophysiological models [47-49] assumes a characteristic relaxation time of 2 ms. This relaxation time represents the time it could take for submembrane shell calcium to relax to the ‘global calcium’ steady state, 40nM in the model. The submembrane shell relaxation time conceptually encompasses: (i) diffusion from beneath the membrane into the rest of the compartment, for example, the spine head, (ii) diffusion into adjacent compartments, for example, from the spine to the adjacent dendrite, (iii) buffering by calcium binding proteins, for example, calbindin and parvalbumin, (iv) uptake into the smooth endoplasmic reticulum by SERCA, and (v) extrusion through the plasma membrane by the Na-Ca exchanger. In addition, calcium extrusion from the cell by the plasma membrane Ca-ATPase (PMCA, calcium pump) is modeled explicitly as a Michaelis-Menten expression.

In order to properly replace submembrane shell calcium with global calcium in the spine and the adjacent dendrite, (i) – (v) are all modeled explicitly. The model by Hernjak et al. [35] assumed instantaneous diffusion in the spine, and included diffusion between the spine and adjacent dendrite. It also included explicit buffering by calbindin and parvalbumin, in addition to calcium uptake by SERCA. That model did not include calcium extrusion from the spine or adjacent dendrite. Here, we add in calcium efflux by including the Michaelis-Menten Ca-ATPase from the Purkinje neuron electrophysiology models [48,49] and a Na-Ca exchanger from a study on cerebellar granule cells [50] (a similar Na-Ca exchanger in a Purkinje neuron model was not available). The Na-Ca exchanger is not electrogenic and simply provides an additional means of calcium extrusion; its kinetics are unchanged in our model. The total concentration of the Ca-ATPase is increased to allow for calcium efflux appropriate for maintaining a physiological steady state calcium concentration in the Purkinje neuron spine. The kinetics for the voltage-gated calcium-activated potassium channels were adjusted to be influenced by global calcium. This reflects results from experimental studies which suggest that BK channels can be activated by global calcium concentrations less than 10 μM [51].

### The ICpeptide study

**Figure 2:**
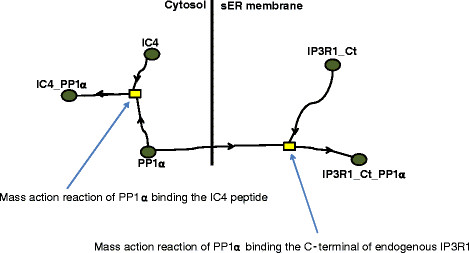
**Schematic for PP1α binding endogenous IP3R1 and a peptide resembling the C-terminal of IP3R1 (IC4).** This corresponds to the ICpeptide study described in the *Methods* section.

The Bezprozvanny lab found that Protein Phosphatase 1 alpha (PP1α) dephosphorylation of IP3R is largely dependent on PP1α binding the very tip of the C-terminal of IP3R1, and that IP3R1 dephosphorylation (increased IP3R1-PP1α binding) decreased the receptor’s sensitivity to IP3 [21,43]. Binding of PP1α to the tip of the endogenous IP3R1 C-terminal and to peptides that resemble the C-terminal of IP3R1 (Figure 2) (here termed ICpeptides, e.g., GST-tagged IC4 in Figure 1a) was added to the model at the ER membrane and in the cytosol, respectively, with mass action kinetics and a stoichiometry of 1. For example:

(1) Kf_IC4^*PP1α^*IC4−Kr_IC4^*IC4_PP1α

where *K*_*f_IC4*_ and *K*_*r_IC4*_ are the forward and reverse rate constants, respectively, for *PP1α* binding the exogenous peptide *IC4*, and *IC4_PP1α* represents PP1α bound to the *IC4* peptide;

(2) Kf_IP3R1_Ct^*PP1α^*IP3R1_Ct−Kr_IP3R1_Ct^*IP3R1−Ct_PP1α

where *K*_*f_IP3R1_Ct*_ and *K*_*r_IP3R1_Ct*_ represent the forward and reverse rate constants for PP1α binding the endogenous tip of the IP3R1 C-terminal, and *IP3R1_Ct_PP1* represents PP1α bound to IP3R1 at the smooth ER (sER). Rate constants are provided in Table 2. It was assumed that PP1α bound the ICpeptides and the endogenous IP3R1 with the same affinity.

**Table 2:** Parameters for new features added to model

**Model Parameter**	**Value**	**Comment**	**Reference**
K_f-IC4_	0.001 uM-1.s-1	Forward rate of IC4 binding PP1α	--
K_r-IC4_	0.0005 s-1	Reverse rate of IC4 binding PP1α	--
K_f-IP3R1-Ct_	10 uM-1.s-1	Forward rate of IP3R1 C-terminal binding PP1α	--
K_r-IP3R1-Ct_	0.0005 s-1	Reverse rate of IP3R1 C-terminal binding PP1α	--
K_f-mutant-IC4_	20 uM-1.s-1	Forward rate of IC4 binding mAtaxin	--
K_r-mutant-IC4_	0.0005 s-1	Reverse rate of IC4 binding mAtaxin	--
K_f-mutant-IP3R1-Ct_	20 uM-1.s-1	Forward rate of IP3R1 C-terminal binding mAtaxin	--
K_r-mutant-IP3R1-Ct_	0.0005 s-1	Reverse rate of IP3R1 C-terminal binding mAtaxin	--
mAtaxin_init	variable	Mutant Ataxin PolyQ Protein	--
PP1α_init	1 uM	Directly binds tip of IP3R1 C-terminal	[43]
IP3R1-Ct_init	1 uM	Tip of IP3R1 C-terminal	--
IC4_init	2 uM	Competitively inhibits PP1α binding	[43]
IC-G2736X_init	2 uM	Associates with polyQ region	[52]
IC10_init	variable	Competitively inhibits polyQ binding	[53]

The C-terminal of IP3R1 can also bind mutant Ataxin. The affinity of the IP3R1 C-terminal tip was taken to be greater for the mutant protein than for PP1α. This was done to incorporate two phenomena into the binding reaction. First, planar lipid bilayer reconstitution experiments from Chen et al. and Liu et al. [6,10] suggest that direct association of the mutant ataxin protein with the C terminal of IP3R1 leads to supersensitivity of the receptor. Second, the binding domain of the mutant protein [52] on IP3R1 overlaps the PP1α binding motif [52] on IP3R1. Mutant protein binding IP3R1 *in situ* (as in cerebellar slices from mouse models that can be used to test simulation predictions; see Discussion) may decrease PP1α interaction with the receptor. These two factors are effectively modeled by assigning a larger affinity for mutant protein binding to IP3R1 than PP1a binding the receptor. Affinities were captured by the rate constants, all available in Table 2.

## Results

Two main categories of *ataxias* in humans and mouse models are considered in this study: those with reduced IP3R1 and those with supersensitive IP3R1 (Table 1). However, some *ataxias* involve both phenomena, adding a layer of complexity. Further, a common component of all these *ataxias* is abnormal calcium metabolism.

### SCA15/16: pathophysiology of IP3R1 insufficiency

**Figure 3:**
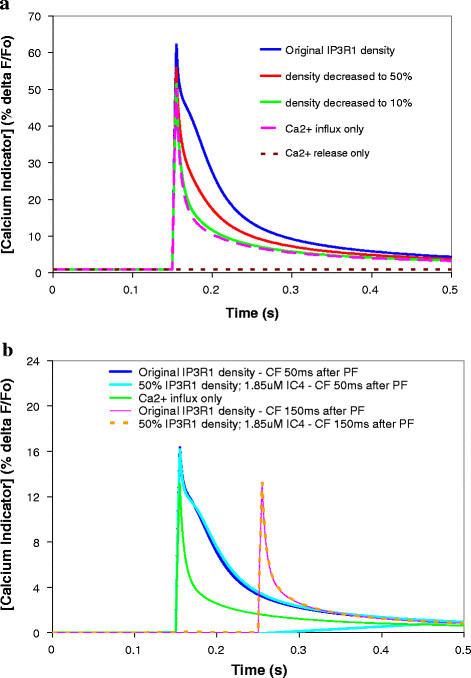
**Increasing IP3R1 sensitivity to IP3 can restore normal calcium response in the context of reduced IP3R1 levels (a model of SCA15/16).a**, Coincident calcium release (from 4 parallel fiber stimuli and a single climbing fiber stimulus) with IP3R1 levels decreased to 50% and 10%. **b**, Wild type levels of supralinear calcium release can be regained in IP3R1 insufficiency by augmenting IP3R1 sensitivity to activation by IP3, using IC4 at a concentration that gives a 1.7-fold increase in IP3R1 sensitivity to IP3. This fold increase in sensitivity is within an experimentally observed range (2-fold) [55]. Fine-tuning of coincidence detection [42] is preserved.

In *ataxias* with reduced IP3R1, e.g., SCA15/16, receptor levels appear to be decreased by varying amounts, even in conditions of heterozygous gene deletion [3,4,14-16,20]. We captured this variability in our model by adjusting the abundance parameter *Jmax* to decrease IP3R1 levels to 50%, 40%, 30%, 25%, and 10% of the original value. Figure 3a shows the supralinear calcium response with simulated wild type IP3R1 levels (labeled as ‘Original IP3R1 density’) when the Purkinje neuron spine is stimulated by a single climbing fiber at 0.15 s (labeled as ‘Ca2+ influx only’), by 4 pulses from a single parallel fiber at 0.1 s (labeled as ‘Ca2 release only’), or by coincident stimulation by 4 pulses from a parallel fiber at 0.1 s and a climbing fiber at 0.15 s. The simulation uses Magnesium Green as the calcium indicator (K_d_ = 19 μm) that fluoresces more brightly when bound to calcium [35,44]. The figure indicates that the coincident calcium transient is greater than the sum of the individual calcium transients from stimulation of only the climbing fiber or only the parallel fiber [35,42,54]; in our simulations, calcium release alone produces a barely perceptible increase in free Ca^2+^, but when it occurs together with calcium influx, the free Ca^2+^ increase more than doubles compared to influx alone. The figure also shows that halving the density of IP3R1 (as in heterozygous mouse models and individuals) severely reduces supralinear calcium release. Thus, the model can be used to investigate the heterozygous human *ataxia* SCA15/16.

### Application of ICpeptide to adjust sensitivity in SCA15/16

**Figure 4:**
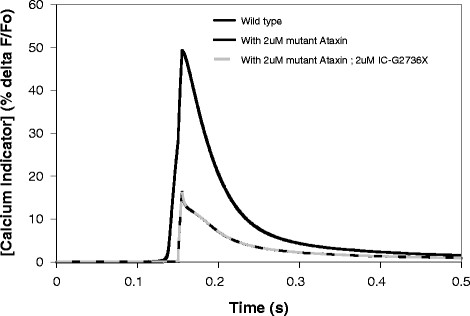
**Simulated application of another ICpeptide in a model of SCA1/2/3 can restore normal calcium release.** Hyperactive calcium release in the presence of mutant Ataxin can be overcome by application of IC-G2736X, which resembles the polyglutamine binding domain in the C-terminal of IP3R1, and was developed by Tang et al. [52]. Note that the dashed light grey line (representing restoration of normal calcium release by application of ICpeptide in the context of a mutant Ataxin model) directly superimposes on the solid black line (which represents wild type).

Modeling results from Hernjak et al. [35] showed that increasing the sensitivity of the IP3R1 by a factor of 10 did not compensate for a decreased the level of IP3R1 to 10% of the wild type value. They argued that the normally low sensitivity of IP3R1 to IP3 was required to assure that calcium release is confined to the activated dendritic spine while the normally high level of IP3R assures robust calcium release despite the low sensitivity. This result is consistent with the finding in Figure 3a, which shows that the calcium transient with 10% IP3R1 abundance is almost entirely due to calcium influx through voltage-gated calcium channels. Very little of the calcium transient is due to calcium release from the ER, through IP3R. It allows us to suggest that in Purkinje cells, with have much lower sensitivity of IP3R to IP3 than in other cell types, decreasing receptor density to 10% of the wild type levels (which amounts to replacing Purkinje neuron IP3R with levels found in other cell types) is as good as having no IP3R at all. Figure 4 shows that increasing sensitivity in the wild type context (by adding mutant Ataxin-2 to the model with normal IP3R levels) disrupts the IP3 response [35]. However, in the context of decreased Purkinje IP3R abundance, augmented sensitivity (whether comparable to other cell types or greater) could potentially restore normal IP3 response if the IP3R1 is not lowered as drastically as the factor of 10 used in the Hernjak [35,54] model.

To assess this, we simulated an additional system (see Table 2) that has been shown experimentally to regulate IP3R1 sensitivity: the phosphorylation state of IP3R1. Tang et al. discovered that dephosphorylation of wild type IP3R1 by PP1α profoundly reduced the receptor’s sensitivity to IP3 and decreased IP3R1-mediated calcium release [43]. Indirect binding occurs through AKAP, an adaptor protein that links IP3R1 to both PKA and PP1αpha by binding to the *LIZ* domain in IP3R1 (see Figure 1a). Tu et al. showed that most association of PP1α, the major dephosphorylator of IP3R1, to the receptor occurs via direct binding at the C-terminal of IP3R1 [21] (see Figure 1a). Tang et al. created GST-IC4, a competitive peptide that resembles the tip of the C-terminal of IP3R1 (36 aa, Q2714-A2749) [43](Table 3). This peptide binds PP1α, preventing its direct association to the C-terminal of IP3R1 (see Figure 1a). They found that dephosphorylation of IP3R1 could be reduced by more than twofold by administering GST-IC4. In the model, we simulated treating Purkinje neurons of SCA15/16 mice with GST-IC4 to see if the resulting increase in IP3R1 sensitivity to IP3, could augment IP3R1-mediated calcium release. Figure 3b shows that as IC4 increases, the resulting decreased association of PP1α with IP3R1 increases the sensitivity of the receptor for IP3 and restores normal calcium transients. In the figure, a 1.7-fold increase in IP3R1 sensitivity corresponds to 1.85uM of IC4 needed to restore normal calcium response when IP3R1 levels are decreased by 50%. This suggests that an experimental study of GST-IC4 treatment in mouse models of SCA15/16 could show normalized IP3 response with a fold increase in sensitivity already demonstrated by Tang et al. in *in vitro* studies [43]. However, as levels of IP3R1 go lower and lower, the fold increase in sensitivity needed to restore normalcy increases nonlinearly (data not shown). In the model, IP3R1 levels could be decreased only as far as to 25% and still be able to restore normal calcium with a fold increase in sensitivity that is experimentally attainable.

**Table 3:** Suggested use of ICpeptides relevant to this study

**ICpeptide**	**Base pairs**	**Minimal binding region of target**	**Use suggested by this study**	**Reference**
IC4	Q2714-A2749	R2731-G2736	*in vivo* competitive inhibition of PP1α	(Tang et al., 2003b,
			with reduced levels of IP3R1	Tu et al., 2004)
IC-G2736X	D2590-G2736	F2627-G2736	*in vitro/vivo* competitive inhibition of mutant Ataxin	(Tang et al., 2009)
			with supersensitive IP3R1	
IC10	F2627-A2749	F2627-G2736	possible alternative to IC-G2726X	(Tang et al., 2009)

The simulations were repeated with the climbing fiber stimulus applied 150 ms after the start of the train of the PF stimuli, unlike the previous results that have a timing difference of 50 ms. A 150 ms timing between the CF and PF stimuli falls outside the optimal time window suggested by both experiments [44,56] and modeling [42]. Figure 3b shows that with this 150 ms time difference, supralinear calcium release is lost in the wild type case, as well as in the heterozygous case. This suggests that restoration of normal calcium release in the model does not alter fine-tuning of coincidence detection suggested by Brown et al. [42]. Thus, increasing IP3R1 sensitivity could be explored as a therapeutic tactic in SCA15/16 mouse models, e.g. IP3R1^delta18/delta18^ or IP3R1^+/−^ mice.

### SCA2: application of ICpeptide in *ataxias* with pathologically increased sensitivity

Tang et al. also found that the huntingtin binding site on IP3R1 is immediately upstream of the PP1α-binding motif on the tip of the IP3R1 C-terminal [52]. The predicted minimal region on IP3R1 for huntingtin polyglutamine repeat binding (F2627-G2736) actually overlaps a small portion (5 aa) of the minimal PP1-binding motif (R2731-A2749)[52]. The huntingtin protein binding IP3R1 may therefore physically occlude or partially hinder binding by PP1αlpha *in vivo* and *in situ*. This would reduce PP1αlpha dephosphorylation of the receptor, leaving it in a hyperphosphorylated and therefore supersensitive state. A similar mechanism should occur with mutant Ataxins in SCA2 [10] and SCA3 [6] and also SCA1 [8,10,57], given that the mutant Ataxin and huntingtin proteins all have similarly expanded CAG repeats.

Figure 4 shows that adding 2 μM mutant Ataxin, e.g. mutant Ataxin-2 in SCA2, to the wild type model triples the increase in simulated fluorescence of the calcium indicator. Furthermore, the IP3R1 supersensitivity allows calcium concentration to start rising even before coincidence with calcium influx. In addition, we simulated the application of a different ICpeptide in our model. This ICpeptide, IC-G2736X (D2590-G2736)(Table 3), was also created by Tang et al., and encompasses the part of IP3R1 that encodes the minimal binding region for the expanded polyglutamine repeats in huntingtin, and likely the mutant ataxin proteins [52]. Further, Tang et al. used a β–galactosidase assay to show that IC-G2736X strongly associates with the mutant polyQ huntingtin [52]. In fact, they also used viral infection to insert IC10 into striatum of ataxic HD mice, and observed consequent stabilized calcium signaling, as well as neuroprotective effects [53]. In our model, IC-G2736X competitively binds the mutant Ataxin protein and blocks mAtaxin from binding endogenous IP3R1. The normal sensitivity of IP3R1 to activation by IP3 is therefore restored, and IP3R1 is no longer supersensitive. Figure 4 shows the restoration of normal supralinear calcium release. This result allows us to predict that experimental application of such an ICpeptide in cerebellar slices from mice with *ataxias* associated with supersensitive IP3R1 and increased calcium release may similarly restore normal calcium response. We expect that various concentrations of ICpeptide could be needed to normalize IP3R1 sensitivity and consequently calcium release in SCA1, SCA2, or SCA3, based on the degrees to which IP3R1 sensitivity to IP3 is pathologically increased. This could depend on the concentration of the mutant protein in the spine, as well as other unknown factors.

### SCA1 (& SCA3): compensation versus pathology

Although expression of IP3R1 and other calcium signaling proteins is reduced in SCA3 mice [7] and in SCA1 mice and humans [9,11], reduced calcium release is not experimentally observed in either disease. It is therefore not understood what role reduced IP3R1 plays in the pathology of these polyQ diseases. Figure 3 suggested that in SCA15/16 and other *ataxias* with reduced IP3R1 abundance and consequently reduced calcium release, therapeutic strategies to increase IP3R1 sensitivity to activation by IP3 could restore normal calcium release. This begs the question of whether, conversely, the downregulation of IP3R1 in *ataxias* with supersensitive IP3R1 could partially or wholly restore normal calcium response. A glimpse into a compensatory effect could lie in examining actual experimentally observed relative expression levels in SCA1 [9,11], and using those observed levels in the model with supersensitive IP3R1. This allows the modeling and simulations to be driven by experimental observations. Accordingly, a 1.8-fold decrease (approximated from Lin et al. and Serra et al.) [9,11] was simulated in the model for SCA1 pathology.

**Figure 5:**
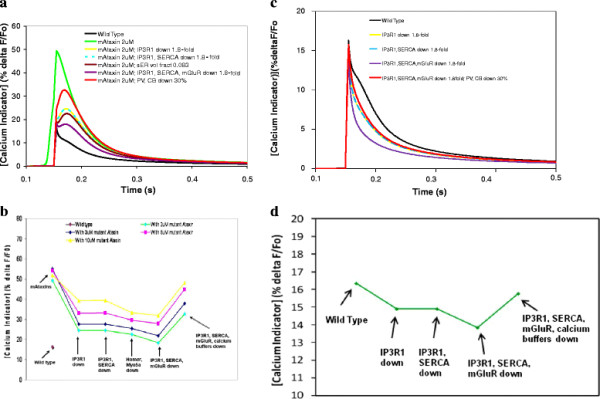
**Downregulation of IP3R1, SERCA, mGluR, and Homer/MyoVa appears to be compensatory, but downregulation of calcium buffers may contribute to pathology.a** &**c**, Supralinear calcium release in wild type and with mutant Ataxin and with each molecule downregulated cumulatively, assuming supersensitive IP3R1 (**a**) or normosensitive IP3R1 (**c**). For the red curve, the calcium buffers PV and CB are downregulated by 30% in addition to at least a 1.8-fold decrease in concentration for IP3R1, SERCA, and mGluR. **b** &**d**, The compensation trend plotted for various potential concentrations of mAtaxin, showing the inverse relationship between the extent of compensation and the relative concentration of mAtaxin in the spine, assuming supersensitive IP3R1 (**b**) or normosensitive IP3R1 (**d**).

Figures 4a and 4b show that tuning down IP3R1 decreases the hyperactive supralinear calcium release to levels closer to wild type. This suggests that experimentally observed reduced abundance of IP3R1 may partially compensate for supersensitivity in SCA1 mice. However, other calcium signaling molecules were also downregulated by at least 1.8-fold [9,11]. To determine the individual effect of the downregulation of some of the molecules, each molecule was sequentially downregulated. Tuning down SERCA in addition to IP3R1 has no additive effect. Myosin Va (MyoVa) and Homer 3 also are decreased [9,11]. MyoVa and Homer 3 are thought to potentially (i) guide sER into the spine, and (ii) anchor sER in the spine, respectively [58,59]. Decreasing sER volume in the spine by 1.8-fold in the *ataxia* model reduces the hyperactive calcium release to levels similar to tuning down IP3R1 or IP3R1 and SERCA. Although the correlation between Homer and MyoVa expression and sER volume fraction is probably not linear, it is remarkable that reducing the volume fraction by 1.8-fold gives a result that is close to directly tuning down IP3R1 and SERCA, which reside on the sER. Given the critical importance of IP3R1 on the sER in normal Purkinje neuron function, reduced volume fraction of spine sER could contribute to the pathophysiology of IP3R1-associated *ataxia*. However, in the context of IP3R1 supersensitivity, Homer and MyoVa downregulation could be compensatory. Finally, tuning down mGluR (by tuning down pulsatile IP3 production) in addition to SERCA and IP3R1 further decreases hyperactive calcium release, so that the resulting supralinear calcium release in the ataxic model is even closer to wild type. This is remarkable, suggesting that mGluR downregulation [9,11] is also compensatory, and additive to IP3R1 downregulation. Not surprisingly, Figure 5b suggests that the greater the concentration of mutant Ataxin, the less the compensation achieved by tuning down IP3R1, SERCA, mGluR, and Homer.

Expression of calcium-binding proteins, parvalbumin and calbindin, are also experimentally observed to be reduced [9,11]. It is not known whether this contributes to pathology or compensation. Calbindin knockout mice crossed with SCA1 mice worsens the phenotype [9]. This suggests that low levels of these buffer proteins could accelerate pathology. It is possible that normal or high levels of buffer proteins may help to maintain homeostasis in the presence of supersensitive IP3R1 early on in the SCA1 disease, then, when their expression is reduced, homeostasis is lost. Modeling results support this suggestion. Tuning down the calcium buffers parvalbumin and calbindin in the model makes supralinear calcium more robust than with down regulation of IP3R1, mGluR and SERCA alone (Figure 5a,b). Accordingly, the downward trend in the peak concentration of calcium is reversed with down regulation of calcium buffers. This is consistent with studies in Purkinje cells from parvalbumin and calbindin knockout mice that are ataxic [60,61], where peak amplitudes of calcium transients evoked by climbing fiber stimulation were double those in wild type mice [62]. Notably, it has been suggested that because reduced expression of buffer proteins is observed in SCA1 mice before onset of symptoms, the timing of buffer protein downregulation may help determine the onset of symptoms [63,64].

Notably, Liu et al. report that mutant SCA1 pathologically associates with IP3R1 [10], as do SCA2 and SCA3 [6,10], suggesting a common supersensitive IP3R1 pathology, but they do not actually measure or report changes in IP3R1 sensitivity. Thus, we thought it was important to consider changes in calcium resulting from signaling protein downregulation, even if hyperactive IP3R1 is not assumed. Figures 5c and 5d illustrate that calcium responses resulting from IP3R1 and other calcium signaling protein downregulation, but with normal IP3R1 sensitivity, are less robust than wild type. This indicates that downregulation of these proteins in the absence IP3R1 supersensitivity does not appear consistent with the elevated calcium response relative to wild type, as observed in SCA1 mice [8]. The model therefore supports the hypothesis of IP3R1 supersensitivity in SCA1.

### Influence of calcium on voltage-gated calcium-activated potassium channels

To better understand the interplay between calcium signaling and electrophysiology, we investigated the relationships between calcium influx, IP3R1-induced calcium release, and the voltage-gated calcium-activated potassium (K_Ca_) channels. Our model includes the BK channel (‘big’ conductance calcium-activated voltage-gated potassium channel) and IK (intermediate conductance calcium-activated voltage-gated potassium channel) channels. Since more is known about the BK channels, we focused our study design and interpretation on these channels (see schematic in Figure 1b). The BK channel in the cerebellum is thought to contribute to repolarization of membrane potential transients in the dendrites, particularly *calcium spikes* (transient depolarization of membrane potential in spines and dendrites) resulting from the opening of voltage-dependent calcium channels in the dendrites [49]. In addition, experimental results by Sausbier et al. using ataxic BK knockout mice suggest that these channels are also important for the afterhyperpolarization of action potentials at the soma [65]. BK channels contribute to precision timing, and are thus important for normal firing of the cerebellar Purkinje neurons [65-70].

IP3R1 interacts closely with the BK channel, in glioma cells [71]. The BK channels seem to be in lipid rafts in the plasma membrane apposed to the sER. It is thought that in other cell types, including some neurons, the BK channels may form physical complexes with various plasma membrane calcium channels, bringing them within a few nanometers of the calcium channel pores [72]. We suspect that in the cerebellar Purkinje neuron spines, BK *activity* is also closely linked with the IP3R1-mediated calcium release. This idea is supported by direct activation of the BK channel by IP3R1-mediated calcium release in pyramidal neurons [73] and cereberal artery smooth muscle cells [74]. We therefore set out to investigate possible activation of BK channels (and IK channels) by IP3R1-mediated calcium release.

**Figure 6:**
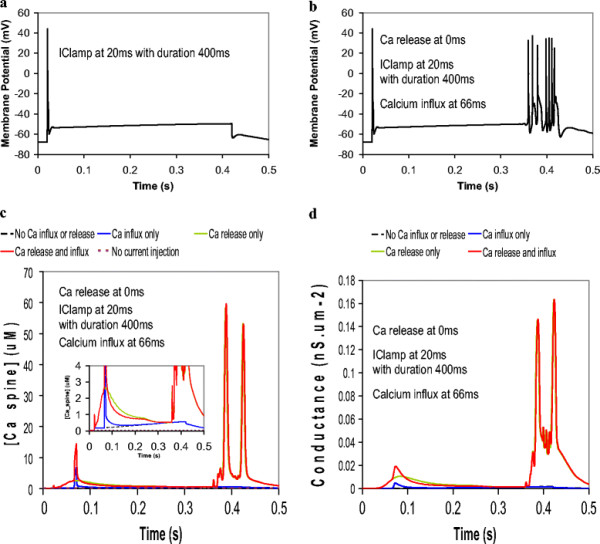
**Membrane potential response to a 400 ms current injection at the soma is altered by biochemical calcium transients in eleven adjacent spines.a**, Membrane potential response at the soma in response to a 400 ms 2 nA depolarizing current injection at the soma. **b**, Action potentials at the soma in response to a depolarizing current injection at the soma superimposed with PF-induced calcium release (20 ms before the start of the current injection). **c**, Corresponding calcium transients at the spine with an *inset* magnifying the lower amplitude responses. **d**, Corresponding changes in BK channel conductance at the spine. Note that in **c** and **d**, the solid red and green lines directly superimpose beyond 0.3 s.

In the model, we allowed calcium released from the sER in eleven spines to activate the voltage-gated K_Ca_ channels. A group of eleven spines represents a small portion of a dendrite branchlet. (The Purkinje neuron has ~ 14 spines per μm^2^ (Harris and Stevens, 1988)). Figure 6a shows that a 20 nA current injected at the soma for 400 ms gives a single sodium action potential spike, followed by a gradual incline. When calcium influx due to CF stimulation is applied 40 ms after the start of the current injection, an identical result is obtained (data not shown). When calcium release due to PF stimulation is applied 20 ms before the start of the current injection, a single sodium action potential spike is obtained, followed by the gradual incline, but then terminates with a short action potential train (Figure 6b). These action potential oscillations are similar to tonic firing oscillations observed experimentally [65]. An identical result is obtained when calcium influx is also added in 40 ms after the start of current injection. Thus in contrast to Figures 2 and 3, where calcium release is stimulated in only one spine, the transient calcium influx may not significantly enhance the overall effect of calcium release in 11 spines. This point is corroborated by examination of the corresponding calcium transients shown in Figure 6c. With PF stimuli only, in the absence of current injection, the Ca transient is minimal. With current injection at the soma, in the absence of biochemical signaling, there is a significant calcium transient at the level of the spines, corresponding to that first membrane potential spike. During that spike, sodium channels open at the soma, and calcium channels open throughout the dendrite. That allows calcium to rush into the cell. The calcium concentration then gradually rises, corresponding to the membrane potential incline. With PF stimuli preceding current injection onset by 20 ms, IP3 is already bound to IP3R1 when calcium rushes in through voltage-gated channels in the spines (in response to the depolarization of the membrane during current injection). This calcium also binds to IP3R1 on the sER. Thus, we have IP3 and calcium bound to IP3R1, yielding coincident activation of the receptor. In this case, coincidence detection is not of PF and CF stimuli, but of PF stimuli and current injection. IP3R1 can therefore serve as a coincidence detector for any activation of the Purkinje neuron that leads to changes in intracellular calcium concentration and increases in IP3 concentration. The figure shows that the resulting calcium transient is supralinear. With the CF stimuli only, a sharp calcium transient is obtained. When the CF stimulus is combined with current injection, the calcium transient is simply additive. This is because neither CF stimulus nor current injection provides IP3, which is necessary for coincidence detection at IP3R1. The corresponding modulation in the conductance of the BK channels is shown in Figure 6d. The figure indicates that BK channel conductance begins to rise almost immediately after either calcium release or calcium influx or both. Not surprisingly, the supralinear calcium transients give the biggest increases in conductance of the BK channels. The influence of the calcium transients on BK channels alters the membrane potential of the Purkinje neuron. The figure shows that with the calcium release from PF stimulus only, termination of the current injection results in a short train of action potentials. As with the membrane potential in Figure 6a, this spike does not occur in the absence of the PF stimulus. With supralinear calcium release activating BK channels, this also gives a termination spike that is identical to that obtained with calcium release only. CF stimulus alone does not alter the membrane potential.

**Figure 7:**
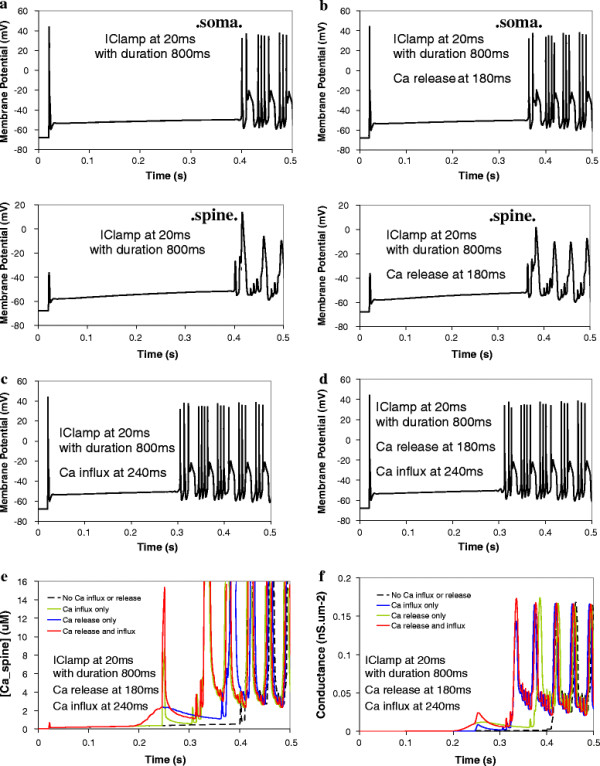
**The onset of action potential oscillations is altered by calcium transients in eleven adjacent spines, with an 800 ms current injection and a different timing sequence between PF and CF stimuli.a**, Membrane potential oscillations at the soma *(top panel)* and the spine *(bottom panel)* in response to a 800 ms depolarizing current injection at the soma (no calcium influx or release). **b**, Membrane potential oscillations at the soma *(top panel)* and at the spine *(bottom panel)* in response to a depolarizing current injection at the soma superimposed with calcium release only (160 ms after the start of the current injection). **c**, Membrane potential oscillations at the soma in response to a depolarizing current injection at the soma superimposed with calcium influx only (220 ms after the start of the current injection). **d**, Membrane potential oscillations at the soma in response to a depolarizing current injection at the soma superimposed with both calcium release and influx. **e**, Corresponding calcium transients at the spine. **f**, Corresponding changes in BK channel conductance.

In Figure 7, current injection commences significantly before both the PF and CF stimuli: PF stimulus occurs 160 ms *after* current injection, but still occurs 60 ms (within the experimentally observed time window [56]) before the CF stimulus. In addition, the current injection now lasts for 800 ms, as opposed to 400 ms in Figure 6. Figure 7a shows that in the presence of a prolonged current injection, a train of action potentials can be induced in the absence of any calcium increase. Consistent with Figure 6, with PF stimulus only, action potential spikes at the soma appear earlier (Figure 7b, *top panel*) than in the absence of the resulting biochemical calcium transient (Figure 7a, *top panel*). The bottom panels in Figures 6a and 6b show the corresponding calcium potential spikes at the spine, with similar results. These calcium spikes occur once a depolarization threshold is reached in the dendrites and spines, in response to high frequency repetitive firing of the Purkinje neuron (corresponding to high amplitude current injection) [49]. The resulting change in membrane potential in the dendrites and the spines propagates to and is reflected at the soma as the transient plateaus that occur in the midst of the sodium spikes. The resulting calcium transients are similar to those in Figure 6, in that superposition of parallel fiber stimulus and current injection gives supralinear calcium release, and further superposition with CF stimulus gives a calcium transient that is additive (Figure 7e). Also similarly, the supralinear calcium transient from calcium release and current injection leads to earlier calcium signal oscillations than in the absence of the biochemical calcium signal (Figure 7e). Interestingly, the calcium obtained from CF stimulus superimposed with current injection results in earlier membrane potential spiking (Figure 7c) than the supralinear calcium release from PF stimulus and current injection superposition (Figure 7b). Virtually identical results are obtained with coincident CF and PF activation superimposed with current injection (Figure 7d) as in Figure 7c. The calcium release portion of the calcium signal falls off more quickly upon superposition of CF and PF stimuli than supralinear calcium release from PF stimulus alone (Figure 7e). This can be attributed to the IP3R1 bell-shaped curve response to calcium concentration [75], where the calcium concentration rises sufficiently during combine Ca^2+^ influx and current injection to significantly populate the inhibited state of IP3R1. Since the calcium response falls off during this sustained potion of the overall Ca^2+^ signal (Figure 7e), the BK conductance also falls off (Figure 7f), thus affecting the membrane potential. Figure 7 suggests that the overall contour of the calcium signal and the resulting K_Ca_ conductances changes may underlie the effect of calcium on membrane excitability.

Taken together, these results suggest that in Purkinje neurons, coincident activation of the Purkinje spine by current injection at the soma and IP3R1-mediated calcium release with or without concurrent CF stimulus can alter membrane excitability. The results indicate that K_Ca_ activity increased by calcium release led to earlier action potential spikes than in the absence of biochemically induced calcium transients. This is reminiscent of a study by Sausbier et al. [65] in which control mice with wild type levels of BK activation showed more spontaneous discharge relative to ataxic BK^−/−^ mice. This is presumably due to another important result from that study: BK activity generates afterhyperpolarization of sodium (action potential) spikes. Thus, augmented BK channel activity polarizes the plasma membrane and increases excitability by more quickly resetting the sodium channels.

Computational models that combine the cell biology of calcium signaling with membrane electrophysiology involving calcium channels and calcium-activated ion channels can reveal emergent properties that are not observed in models of purely biochemical or purely electrophysiological models. The models that are presented in this work can be useful for studying these emergent properties in normal physiological Purkinje neurons, as well as in *ataxia*. Future studies can be developed to study spontaneous Purkinje neuron firing rate or precision of spike timing and interspike intervals in various classes of *ataxias* with mutations in genes that code for calcium channels [9,76-78] and potassium channels [65,79], as well as IP3R1 or related proteins (including SCA1, SCA2, SCA3, SCA15/16) [3,4,6,7,9-16].

## Discussion

We determined that the Virtual Cell modeling environment [36] can be used to study cellular dynamics associated with disease states, particularly spinocerebellar *ataxias* with altered IP3R1 abundance or sensitivity or both. We modified a quantitative multicompartmental model recently developed in Virtual Cell to investigate the coupling of detailed biochemistry of IP3 and calcium signaling with membrane potential. Results show that computational modeling allows us to simulate the behavior of calcium dynamics and membrane potentials in neurons at the level of the spine and of the soma, in response to various stimuli. Our models include a myriad of ion channels and molecules important for normal Purkinje neuron function. They merge features of IP3 production, phosphoinositide and calcium signaling with conceptual representations of parallel fiber and climbing fiber stimulation, local PIP2 synthesis and sequestration, PIP2 hydrolysis, calcium influx, coincident detection at IP3R1, modulation of IP3R1 abundance and sensitivity, IP3R1-mediated supralinear calcium release, and components of membrane electrophysiology into comprehensive models that can have compartmental and spatial counterparts.

Results suggest that ICpeptides may be used to modulate calcium release in various IP3R1-associated *ataxias* (*ataxia*s in which IP3R1 is less abundant, e.g., SCA15/SCA16 as well as SCA1 and SCA3, or more sensitive than in wild type mice or unaffected individuals, e.g., SCA2 and SCA3 and likely SCA1). Simulations predict that pathophysiology associated with decreases in IP3R1 abundance can be overcome by adjusting IP3R1 sensitivity to activation by IP3, and that targeting PP1α can restore normal calcium transients. Various concentrations of ICpeptide may be needed to increase IP3R1 sensitivity in SCA15/SCA16, based on the levels of reduced abundance. Similarly, various concentrations of ICpeptide could be needed to normalize IP3R1 sensitivity and consequently calcium release in SCA1, SCA2, and SCA3, based on the degrees to which IP3R1 sensitivity to IP3 is pathologically increased (dependent on the concentration of mutant protein and the relative balance with PP1α).

This project complements results by Hernjak et al. from a study on Purkinje spine calcium signaling that considered the importance of low sensitivity and high abundance of IP3R1 suggested by experiments [35]. Findings from that study suggested that increasing IP3R1 sensitivity to IP3 could not rescue calcium release in the context of low IP3. However, our results show that increased sensitivity *can* restore normal IP3 response *if the abundance is not too low.* This provides further insight into the roles that IP3R1 *abundance* and *sensitivity* play in normal cerebellar functioning and coincident detection at IP3R1. IP3R1 abundance and sensitivity can each function as the primary pathology in IP3R1-associated *ataxias* or can serve to partially or wholly compensate the effects of pathology. Other important conclusions from the study by Hernjak et al. were that the low IP3R1 sensitivity observed in wild type Purkinje neurons relative to other cell types may be important to restrict biochemical signals and synaptic plasticity to one spine, and that the high IP3R1 abundance in Purkinje neurons is important for ensuring generation of a robust calcium signal in an individual spine. The current study confirms that the balance of IP3R1 abundance and sensitivity is critical for obtaining robust, but not hyperactive, calcium transients. It is possible that in *ataxias* with reduced abundance of IP3R1, some spines will not generate robust calcium signals, while in *ataxias* with supersensitive IP3R1, stimulation signals may not be confined to single spines. This could affect both compartmentalization of biochemical signals, and the influence of the biochemistry on membrane electrophysiology. Similarly, therapeutically increased IP3R1 sensitivity (by application of an appropriate ICpeptide) could potentially lead to spillover of calcium into adjacent spines, if sensitivity is not finely tuned. Modeling could thus be useful for helping to determine the adequate levels of ICpeptide to be administered experimentally.

Experimental observations in SCA1 mice appear to be paradoxical [30] (Table 1). The expression of various molecules (for example, IP3R1, Homer, and SERCA) involved in glutamatergic calcium signaling and IP3R1-mediated calcium release is reduced in mice and humans with SCA1 [9,11]. However, IP3-induced calcium release is hyperactive in SCA1 mice [8]. Insight may be gained by examining the pathology of SCA2 and SCA3. Liu et al. [10] and Chen et al. [6] found that mutant Ataxin-2 and Ataxin-3, respectively, bind the C-terminal of IP3R1 and increase the receptor’s sensitivity to activation by IP3. This corresponds to hyperactive IP3-induced calcium release in both SCA2 and SCA3. Above normal numbers of CAG repeats in SCA1, SCA2, and SCA3 give each respective mutant ataxin protein a toxic gain of function that disrupts calcium homeostasis in neurons. Thus, there may be a common mechanism underlying some of the pathology of the three polyQ *ataxias*. This mechanism could be binding of the polyQ-expanded protein to the C-terminal of IP3R1, thereby increasing the receptor’s sensitivity to IP3. This mechanism may be extendable to a large variety of polyglutamine diseases. In fact, Liu et al. reported that the mutant Ataxin-1 protein and polyQ-expanded atrophin-1, the protein mutated in Dentatorubral-pallidoluysian atrophy (DRPLA), both associate with the C-terminal of IP3R1 [10], though IP3R1 supersensitivity has not yet been assessed experimentally in either disease. Of note, a prominent feature of DRPLA is cerebellar *ataxia*[80-83].

The pathological gain of function of IP3R1 sensitivity that destabilizes calcium signaling [84] is also observed in a mouse model of Huntington’s disease (HD), another polyQ disease [18,52,53,85-87]. Lentiviral and adenoviral infection of affected cultured medial striatal neurons or in the striatum of the ataxic HD mice themselves with the IC10 peptide lead to reduced brain atrophy and improved motor coordination, respectively [53]. Perhaps similar genetic targeting of Purkinje neurons in the cerebellum of existing mouse models with IC-G2736X could mitigate the pathology of SCA1, SCA2, and SCA3. This could first be tested in *in situ* experiments using cerebellar slices from existing mouse models, in which mutant ataxin1 binding IP3R1 directly upstream of the PP1αlpha binding site, would be close enough to preclude binding of PP1αlpha.

Reduced gene expression of IP3R1, Homer/MyoVa, mGluR and other molecules in SCA1 may be due to direct pathology due to the effect of the mutant proteins on transcription [7,9]; it could also be due to the tight regulation of calcium homeostasis in the cerebellar Purkinje neuron [30]. Lin et al. suggest that reduced gene expression is a part of the pathology [9], but our model indicates that with exception of the calcium buffer proteins the effect on IP3R1-mediated calcium release is partially compensatory. Using the reduced fold expression observed in SCA1, normal calcium release was restored to varying degrees, depending on how much mutant Ataxin protein was placed in the model. Reduced expression of several calcium signaling genes is also observed in some Purkinje neuron subtypes in plasma membrane calcium ATP-ase (PMCA) knockout mice [20]. This could also have the effect of partial compensation in that mouse model, since PMCA contributes to expelling calcium from the Purkinje neuron cytoplasm. ‘Compensatory pathology’ is thus an interesting characteristic of various *ataxias*, and provides a level of complexity to the study of these neurological disorders. Disruption of calcium signaling and homeostasis, whether due to reduced IP3R1 levels or supersensitive IP3R1, whether in IP3R1-associated *ataxias* or in other *ataxias,* can lead to dysfunction of Purkinje cells, and impaired long-term depression and synaptic plasticity that are involved in learning and memory.

Calcium homeostasis is critical for normal function of Purkinje neurons and is thought to be tightly regulated [30]. Downregulation of parvalbumin and calbindin could therefore lead to a loss of compensation, by destabilizing supralinear calcium release and disrupting any homeostasis achieved by downregulation of other molecules. Furthermore, it has been postulated that normal or high concentration of calcium buffer proteins have a protective role in certain neurons [63]. Vig et al. suspect that the decrease in parvalbumin expression in Purkinje cells from SCA1 patients may reflect alterations in a regulatory biochemical pathway that may be important for neuronal survival [63]. Further, our model suggests that these changes (downregulation of various calcium channels and buffers) alone may not be sufficient to reproduce elevated calcium response as observed experimentally in SCA1 mice, without assuming concurrent supersensitive IP3R1.

Purkinje neurons are largely spared in SCA3 [88], while nerve cells in the pons and substantia nigra are substantially damaged. Chou et al. suggest that although prominent neuronal loss was not found in the cerebellum, the SCA3 mice displayed pronounced ataxic symptoms, suggesting that instead of neuronal demise, mutant Ataxin3 causes neuronal dysfunction and resulting *ataxia*[7]. While Ataxin1 is found in the nucleus, Ataxin 2 and Ataxin3 are cytoplasmic proteins, under normal conditions. However, in brains from patients with SCA3, mutant Ataxin3 accumulates in the nucleus [28,89], as does mutant Ataxin1 [90]. Hence, results from our study of SCA1 could be extended to SCA3 modeling.

Long term feeding of SCA2 and SCA3 mice with dantrolene improved motor coordination and slowed brain atrophy [6,10]. Dantrolene is thought of as a ‘calcium stabilizer’ [6,10,91] and has been shown to inhibit the ryanodine receptor (RYR), which is another calcium channel on the smooth endoplasmic reticulum [92,93]. However, all the targets of dantrolene are not known [94]. The details of the mechanism of action of dantrolene are incomplete [94], though it has been proposed as a possible therapeutic drug for SCA2 [10] and SCA3 [6]. The drug is currently approved to treat malignant hypothermia as a one-time application in response to adverse reaction due to anesthesia [94-96]. It is also used to reduce muscle spasticity in patients with neurological incidents or disorders. However, dantrolene leads to fluid buildup in the lungs, among other adverse effects [94]. Yet, considering the disturbed neuronal calcium signaling observed in polyQ *ataxias*, it is likely that inhibiting or downregulating either or both of the sER intracellular calcium release channels (IP3R1 and RYR) should attenuate IP3R1-mediated supralinear calcium release into the cytosol and thereby alleviate SCA2 and SCA3. This is particularly so, since both the RYR and IP3R1 channels increase in function when directly bound by cytosolic calcium. Therefore the supralinear calcium release cascade initiated by IP3 binding IP3R1 may involve cytosolic calcium also binding RYR to increase calcium release from the sER. Adding RYR to the model in the future will facilitate further study of the signaling interactions among RYR, IP3R1, other calcium channels, and the calcium-activated potassium channels. However, it should be noted that there is evidence that RYR is localized to the dendritic shaft and is excluded from spines [97,98]. Yet, there is also evidence that these physically separate calcium release sites functionally interact [99].

Predictions from our computer model involving SCA15/16 could be compared with novel experiments in an existing mouse model using GST-IC4 to dissociate PP1α from IP3R1 in Purkinje neurons in cerebellar slices from ataxic mice. This could restore normal calcium and membrane potential response in ataxic mice. For such experiments, it would be useful to select a mouse model that: (i) does not completely knock out IP3R1 expression as in the IP3R1^−/−^ knockout mice, but has reduced expression of IP3R1 protein, (ii) shows motor discoordination, (iii) does *not* possess a mutation in neuronal IP3R1 at the preferred site of PKA phosphorylation (Ser-1755) [100], and (iv) does *not* possess an IP3R1 mutation in the carboxyl terminal PP1α-binding site (2731–2749) [43]. For assessing IP3R1 haploinsufficiency in particular, as found in SCA15 and SCA16 patients [3,12,15,16], it would also be useful to select a mouse model with a large heterozygous deletion mutation in IP3R1. The IP3R1 ^+/−^ mice [2,5] fulfill all of these desired features. The IP3R1^delta18/delta18^ mice have also been suggested as a mouse model for SCA15/16 [3]. The *ITPR1*^opt/opt^ mouse model (see Figure 1b) shows reduced IP3R1 protein levels on Western blot, but has a mutation that deletes the preferred PKA phosphorylation site (Ser-1755) [4,100] in neuronal IP3R1, and therefore violates (iv). Given that a very small region of the gene is deleted and that it occurs at such an important regulation site, we suspect that IP3R1 is dysregulated in these mice. As such, we expect that these mice possess cellular pathophysiology resulting from dysregulation in addition to effects of IP3R1 insufficiency. Consequently, it is yet unclear whether results from this study will be easily extendable to calcium signaling and membrane excitability in *ITPR1*^opt/opt^ mice.

Results from our models suggest that IP3R1-mediated calcium release can activate voltage-gated K_Ca_ channels and thereby alter membrane excitability in the Purkinje neuron. This can have implications for *ataxias* that involve disruption of intracellular calcium homeostasis. It is possible that pathological alterations in calcium transients result in pathological activation of BK channels. This could lead to variations in the timing of action potentials and other electrophysiological events in the Purkinje neuron, which controls modulation of neurons downstream of the cerebellum.

## Conclusions

In summary, we created a series of models incorporating biochemistry and electrophysiology that unify observations in various SCAs. These models employ several novel concepts and approaches and provide a framework for the study not only of IP3R1-associated *ataxias*, but of various SCAs involving mutations of other molecules in the model, such as potassium channels [65,79,101,102] and calcium channels [29,76,77,103].

Model results indicate that, in mouse models of various *ataxias* associated with activity of the calcium channel IP3R1, ICpeptides may be used to stabilize intracellular calcium concentration. Further, restoration of normal calcium release in the model does not alter fine-tuning of coincidence detection suggested by Brown et al. [42]. The hypothesis of IP3R1 supersensitivity in SCA1 is supported by simulation results. Even more, IP3R1 downregulation experimentally observed in SCA1 mice may partially compensate for the receptor’s supersensitivity. Homer and MyoVa downregulation are further compensatory. However, downregulation of calcium buffer proteins accelerates pathology. The model demonstrates that IP3-mediated calcium release in the Purkinje neuron could activate voltage-gated K_Ca_ channels, namely BK and IK, and provides insight into the interplay between IP3R1 sensitivity and abundance in the function and dysfunction of the Purkinje cell. Results help to explain experimental findings in mice, and can be used to make predictions for further experiments, which may ultimately be translated to ataxic individuals with reduced IP3R1 protein levels or increased sensitivity. IP3R1 abundance and sensitivity are components involved in calcium signaling, but by no means the only factors involved in the signaling systems of these SCAs.

## Competing interests

The authors declare that they have no competing interests.

## Authors’ contributions

All authors designed and implemented the models and virtual experiments. All authors wrote the manuscript. All authors read and approved the final manuscript.
